# The Medical Society of the County of Erie

**Published:** 1891-06

**Authors:** 


THE MEDICAL SOCIETY OF THE COUNTY OF ERIE held a memorial meeting on Saturday evening, May 16, 1891, at which appropriate action was taken in reference to the decease of Dr. Geo. N. Burwell. The attendance was unusually large, and the remarks made by many of the speakers were feelingly and appropriately delivered. The Society voted to attend Dr. Burwell's funeral in a body, and to spread upon its minutes a tribute to his memory. We expect to publish the proceedings in full in our next number, as the minutes were not received in season to appear in this issue.

